# Cesarean Scar Ectopic Pregnancy: A Case Report

**DOI:** 10.7759/cureus.78312

**Published:** 2025-01-31

**Authors:** Rajalakshmi Srinivasan

**Affiliations:** 1 Obstetrics and Gynecology, Medcare Royal Specialty Hospital, Dubai, ARE

**Keywords:** beta-hcg, cesarean scar ectopic pregnancy, hemorrhage, hysterectomy, transvaginal sonography

## Abstract

Cesarean scar ectopic pregnancies (CSEP) are a rare type of ectopic pregnancy. This condition occurs when a blastocyst is implanted in the scar left by a previous cesarean section (C-section). CSEP can lead to a risk of maternal hemorrhage, which in severe cases can lead to maternal mortality. In recent decades, the incidence of scar ectopic pregnancies has surged significantly, largely due to the increasing rate of C-sections worldwide. Here, we present a case of a 30-year-old third gravida, with a previous C-section performed a year ago, with no living child. The patient presented with a delayed menstrual period and was subsequently diagnosed with ectopic scar pregnancy. Hysteroscopic evacuation of the ectopic pregnancy was performed, preventing progression to the placenta accreta spectrum. In this case, we aimed to establish the potential influence of previous C-sections on the development of ectopic pregnancies. Given the severity of CSEP and its potential to cause maternal mortality, this case highlights the importance of maintaining a high index of suspicion, particularly in women with prior cesarean deliveries. Early detection of abnormal pregnancies is crucial to preventing complications and reducing the need for invasive surgeries.

## Introduction

Cesarean scar ectopic pregnancy (CSEP) is a condition that can be life-threatening. CSEPs are rare, occurring in approximately one in 2000 pregnancies [1]. As the rate of cesarean section (C-section) is rising globally, the incidence of CSEP is likely to increase, which can lead to complications such as abdominal pain, catastrophic hemorrhage, uterine rupture, vaginal bleeding, abnormal placentation, preterm delivery, and even death [1,2]. These complications make CSEP a high-risk condition for pregnant women therefore, early recognition and management of this condition are critical in preventing these severe outcomes.

CSEP is associated with various risk factors which include assisted reproductive technologies such as in vitro fertilization (IVF), sexually transmitted infections, previous C-section deliveries, increased maternal age, tubal ligation, and other uterine procedures like hysterotomy [1,3]. These factors raise the likelihood of scarring, which makes the uterus more susceptible to abnormal implantation.

The possible pathophysiology of CSEP involves a defect in the lower segment of the myometrial wedge or the presence of a small fistula within the scar. This causes the gestational sac to be enveloped on all sides by myometrial tissue [1].

Regular imaging, preferably during the first trimester, is essential to prevent abnormal pregnancies, which pose significant risks to maternal health. However, diagnosing CSEP can be challenging because of non-specific symptoms such as vaginal bleeding and abdominal pain [4], which are common clinical presentations in pregnancy. Early diagnosis before nine weeks of gestation is essential to reduce maternal morbidity [5]. Without appropriate management, it can lead to the loss of fertility and maternal mortality [6].

Here, we present a case of a third gravida diagnosed with a CSEP, with a history of a C-section performed one year earlier. Hysteroscopic evacuation of the ectopic pregnancy was performed. The patient experienced severe hemorrhage, which was effectively managed using Foley tamponade. This case sought to investigate the potential role of C-sections in the development of ectopic pregnancies, emphasizing the need for awareness among patients and clinicians.

## Case presentation

A case of a 30-year-old third gravida with no living child and one abortion (G3P1A1), who had a history of a previous C-section presented with a five-week and five-day delay in menstruation, having a history of regular menstrual cycles. Informed consent was obtained from the patient after which clinical investigations were followed.

Examination

A transvaginal ultrasound (TVS) was conducted in the outpatient department (OPD), which revealed a small gestational sac located anteriorly in the lower uterine segment at the level of the previous cesarean scar. The cervix appeared long and closed, with no gestational tissue present. Laboratory tests revealed a β-human chorionic gonadotropin (β-hCG) level of 7969 mIU/ml on initial presentation.

Imaging

The TVS from the radiologist confirmed cesarean scar ectopic pregnancy at five weeks and four days of gestation. The gestational sac measuring 7.8 mm abutting the lower segment C-section (LSCS) scar was observed and the yolk sac measured 1.3 mm. The myometrial thickness was measured to be 4 mm with no cardiac activity, and a 48-hour follow-up revealed that β-hCG had risen to 10000 mIU/ml.

Differential diagnosis

Differential diagnoses included missed abortion or spontaneous abortion. A wait-and-watch approach was adopted for a week and to report if any pain or bleeding appeared.

A follow-up scan conducted one week later revealed a Type 2 severe grade CSEP. The crown-rump length (CRL) measured 5.3 mm, corresponding to a gestational age of six weeks and two days. The pregnancy was implanted into the LSCS scar, leaving a residual myometrium thickness of 1.9 mm.

Management

Various treatment options were discussed, and hysteroscopic evacuation of the ectopic pregnancy was chosen, taking into account the risk of hemorrhage, the availability of expertise, and the ease of management.

Surgical findings

**Figure 1:**
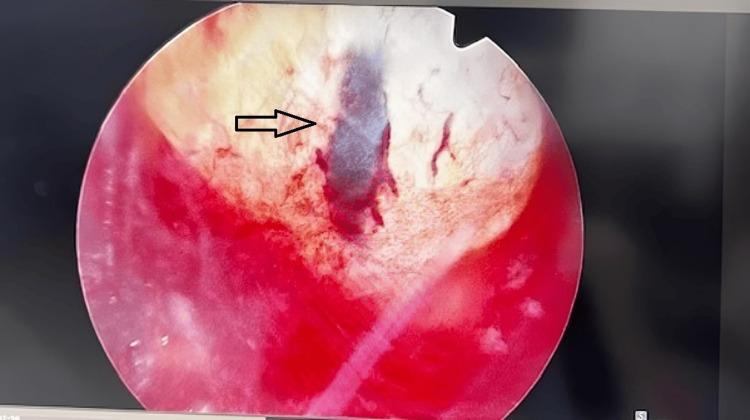
Hysteroscopic image showing the gestational sac firmly implanted within the cesarean scar tissue, surrounded by vascularized structures, prior to surgical removal Gestation sac observed on the anterior wall low segment as soon as the hysteroscope was introduced.

**Figure 2:**
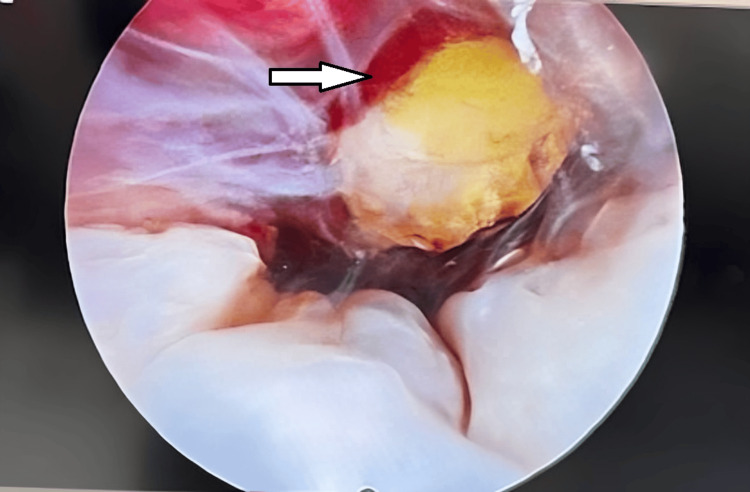
Hysteroscopic view of a gestational sac embedded in cesarean scar tissue with surrounding decidual reaction

**Figure 3:**
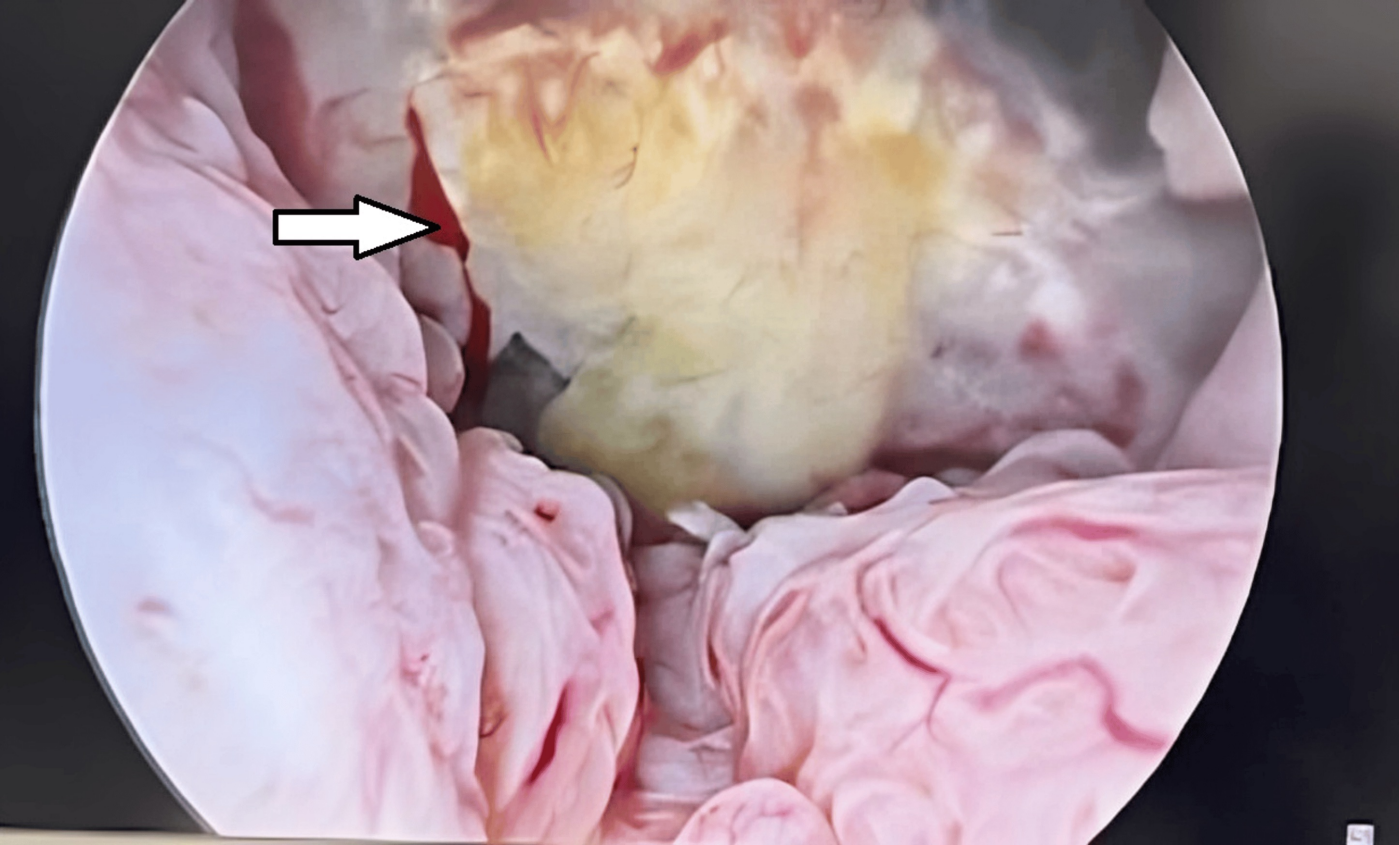
Hysteroscopic image showing a gestational sac implanted in the cesarean scar with visible decidual reaction and vascular congestion

**Figure 4:**
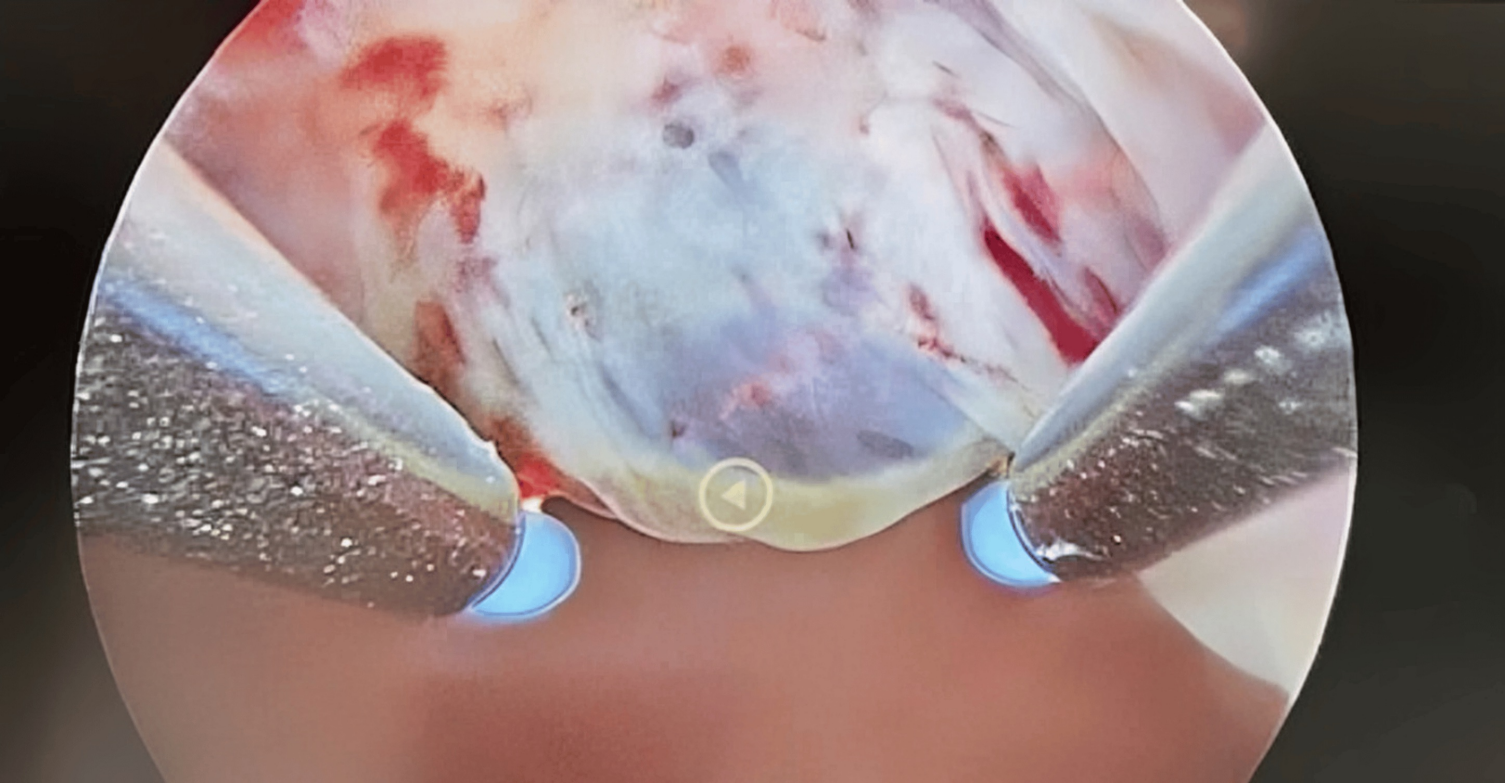
Hysteroscopic image showing the surgical removal of the gestational sac from the cesarean scar site using specialized instruments

**Figure 5:**
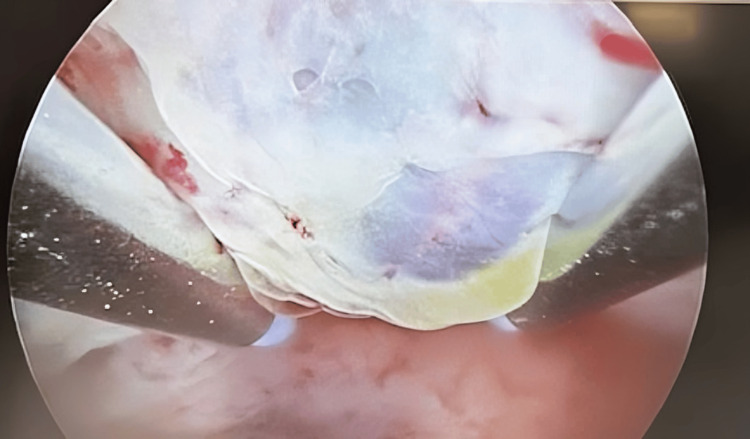
Hysteroscopic image showing the final stages of gestational sac removal from the cesarean scar site, with clear visualization of the surrounding tissue

Hysteroscopy revealed a gestational sac with a good decidual reaction in the lower uterine segment on the right lateral wall at the previous LSCS site (Figures 1-3). The sac was successfully evacuated, and during the course, the patient experienced approximately 500 ml of blood loss, which was managed using Foley’s tamponade (Figures 4, 5).

Post-operative course and follow-up

The patient was discharged on the day after the operation and had an uneventful postoperative recovery period. Given a course of antibiotics, there was no pain and minimal bleeding. The patient re-scan after one week showed a clean uterine cavity. Histopathology reports the presence of products of conception.

## Discussion

CSEP is a rare but serious condition associated with significant maternal morbidity, highlighting the need for increased awareness among clinicians. Early detection and timely intervention are critical in preventing complications such as severe hemorrhage, uterine rupture, and maternal mortality [5].

The exact pathophysiology of CSEP remains poorly understood, but several studies suggest potential mechanisms. Limited vascularization of the lower uterine segment following a cesarean section can impair the healing process, creating a vulnerable scar site prone to trophoblast implantation [2]. This abnormal implantation within the scar tissue disrupts normal pregnancy progression and poses a high risk for maternal complications. Raising awareness and promoting early diagnostic strategies, such as transvaginal sonography and β-hCG monitoring, are essential steps toward improving outcomes in patients with CSEP.

Diagnosis of CSEP

The diagnosis of CSEP primarily relies on imaging techniques such as transvaginal sonography and Doppler ultrasonography, combined with monitoring of β-hCG levels. Emerging research suggests that serum markers may serve as effective diagnostic alternatives to β-hCG in the future [2,5,4]. In alignment with this case, other studies have reported similar presentations, such as a woman with a history of cesarean sections diagnosed with a complex cesarean scar pregnancy [7].

Key risk factors like myometrial thickness and gestational sac diameter are critical considerations, as they independently increase the risk of intraoperative hemorrhage during CSEP management [6].

Influential factors in therapeutic approaches

The choice of intervention for CSEP is influenced by factors such as myometrial thickness and gestational sac size. In this case, the myometrial thickness was 1.9 mm, with a gestational sac measuring 7.8 mm and a yolk sac of 1.3 mm. Lin et al. outlined management guidelines for CSEP based on these parameters. According to American College of Obstetricians and Gynecologists (ACOG) recommendations, suction curettage with hysteroscopy is appropriate for cases with myometrial thickness of 1-3 mm and gestational sac size under 30 mm. For larger sacs or thinner myometrium, transvaginal excision or hysteroscopy combined with laparoscopy may be more suitable. When the sac size exceeds 50 mm, treatments such as uterine artery embolization (UAE), methotrexate, or balloon tamponade are recommended. In this patient, a tamponade was successfully used to control bleeding [8].

Approaches to management

The management of CSEP depends on factors such as the site of implantation, gestational age, and β-hCG levels. For stable patients with β-hCG levels below 5000 mIU/ml, procedures like uterine artery embolization may be considered, though complications like uterine rupture must be considered [2]. Surgical methods remain the most effective approach for removing gestational tissue and repairing uterine defects while preserving fertility. Common techniques include laparoscopy, hysterectomy, and ultrasound-guided vacuum aspiration, with the choice of intervention depending on the surgeon’s expertise and case complexity [2,7].

The management of CSEP often requires a tailored approach based on clinical presentation, patient stability, and available resources. Similar to our case, where hysteroscopy was utilized effectively, studies have emphasized the advantages of surgical intervention in ensuring uterine integrity and minimizing complications. Harpham et al. highlighted that hysteroscopy, while definitive, also has the added benefit of preventing postoperative intrauterine adhesions, a significant concern in reproductive health [9]. Golan et al. further corroborated the importance of surgical precision, noting that hysteroscopic removal of retained products preserves the uterine cavity and prevents trauma, which aligns with the goals of our management strategy [10].

In our patient, the successful evacuation of the gestational sac through hysteroscopy preserved the structural and functional integrity of the uterus, allowing for the potential of future pregnancies. While hysteroscopy is advantageous for direct visualization and reduced risk of retained products, the necessity of adjunctive procedures like hysterectomy in similar cases demonstrates the variability in clinical management based on severity.

Contrasting this surgical approach, Malhotra et al. reported promising results with non-invasive management using systemic methotrexate, particularly in cases with lower β-hCG levels (≤5000 IU/L) [11]. Methotrexate administration, dosed at 50 mg/m², provides a less invasive alternative, with the caveat that patient stability is a critical determinant for its success. This approach aligns with findings from Joshi et al., who demonstrated the feasibility of methotrexate for stable patients without immediate risks of hemorrhage or rupture [2]. While systemic methotrexate is effective in appropriate cases, its limitations include the requirement for stringent follow-up and monitoring of serum β-hCG levels to assess response.

However, there remains no definitive consensus on the ideal management strategy, with over 31 documented treatment modalities for CSEP [12]. This highlights the complexity of the condition, and the importance of individualized treatment planning based on patient-specific factors such as β-hCG levels, myometrial thickness, and gestational sac size.

This case emphasizes the need for a high index of suspicion, timely diagnosis, and expert management to prevent complications associated with CSEP, such as placenta accreta spectrum, which could lead to conditions such as prematurity and severe hemorrhage, and the need to perform complex procedures such as hysterectomy. The patient was managed as a day case with no morbidity and retained the capability for future pregnancies, although the risk of a repeat scar pregnancy remained.

## Conclusions

Early diagnosis in the first trimester is crucial to prevent complications in abnormal pregnancies like CSEP. In this case, timely hysteroscopic evacuation avoided severe outcomes such as hemorrhage or maternal death. These findings emphasize the role of prior cesarean sections and other potential factors in CSEP development. Further research is needed to understand the pathophysiology and improve diagnostic and therapeutic strategies for better outcomes.
